# A circadian clock gene homolog regulates developmental timing and male mating circuitry in *C. elegans*

**DOI:** 10.1016/j.isci.2026.116659

**Published:** 2026-07-02

**Authors:** Shiraz Nir Halber, Eshkar Nir, Shay Stern, Meital Oren-Suissa

**Affiliations:** 1 Department of Brain Sciences, Weizmann Institute of Science, Rehovot 7630031, Israel; 2 Department of Molecular Neuroscience, Weizmann Institute of Science, Rehovot 7630031, Israel; 3 Faculty of Biology, Technion-Israel Institute of Technology, Haifa 3200003, Israel

**Keywords:** *C. elegans*, developmental timing, circadian rhythms, neuronal circuits, CES-2, sexual dimorphism, developmentally timed sleep

## Abstract

Circadian rhythms and sleep are known regulators of neurodevelopment, yet the mechanisms by which they influence neuronal circuit formation remain unclear. In *Caenorhabditis elegans*, postembryonic development comprises four larval stages separated by developmentally timed sleep (DTS) and is regulated by homologs of circadian clock genes. Here, we leverage the well-defined male mating circuitry to investigate how developmental timing and sleep affect neurodevelopment. We found that males exhibit accelerated development with altered DTS patterns and reduced quiescence. Surprisingly, perturbing DTS does not impair male mating, suggesting that developmental sleep is not essential for functional circuit formation in this context. Disruption of the clock gene homolog *ces-2* resulted in delayed and variable development in both sexes. In males, these timing defects were associated with reduced mating abilities and decreased synaptic connectivity within the mating circuitry. Together, our findings support the presence of conserved molecular machinery that coordinates developmental rhythms and provide insight into how such rhythms influence neurodevelopment.

## Highlights

• In *C. elegans*, development time and sleep patterns are sexually dimorphic
• Sleep loss during development does not impair male mating abilities
• The clock gene homolog *ces-2* is required for stereotypical timing of development
• *ces-2* mutant males exhibit reduced mating abilities with altered mating circuitry

## Graphical abstract

**Figure:**
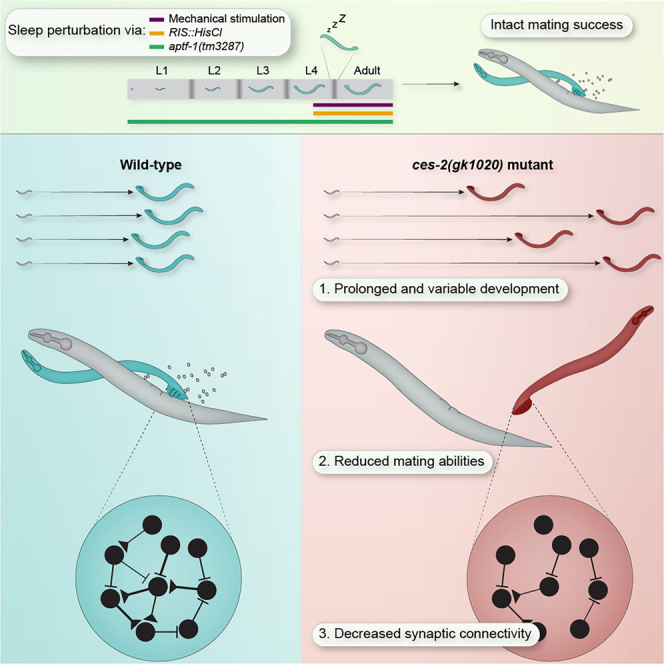

## Teaser

Neuroscience; Behavioral neuroscience; Developmental neuroscience

## Introduction

Biological rhythms are physiological processes that occur repetitively at a certain frequency.^1^ One of the most studied examples is the circadian rhythm, which enables organisms to align their physiology and behavior with the 24-h light-dark cycle.^2^ This rhythm is driven by an internal molecular clock composed of transcriptional-translational feedback loops, in which clock genes and their protein products regulate each other’s expression to generate self-sustained oscillations.^2^^,^^3^ In humans, circadian rhythms emerge during early infancy and regulate many rhythmic processes across the lifespan, such as a daily sleep-wake cycle.^4^ Altered circadian rhythms and sleep patterns are common features of many neurodevelopmental disorders.^5^^,^^6^^,^^7^^,^^8^^,^^9^ For instance, sleep disturbances are prevalent among children with autism spectrum disorder (ASD) and have been shown to correlate with the severity of behavioral symptoms.^10^^,^^11^^,^^12^^,^^13^ Additionally, individuals with attention-deficit hyperactivity disorder (ADHD) often suffer from co-occurring sleep disorders and circadian rhythm alterations,^14^^,^^15^^,^^16^ manifested by delayed sleep onset, altered melatonin secretion, and higher variability of sleep-wake patterns.^17^^,^^18^^,^^19^

A growing body of evidence indicates that early-life sleep loss has long-lasting effects on brain structure and behavior,^20^^,^^21^^,^^22^^,^^23^ and sleep has been implicated in neurodevelopmental processes such as neurogenesis, myelination, and synaptic plasticity.^24^^,^^25^^,^^26^^,^^27^^,^^28^^,^^29^ While vertebrate studies link early-life circadian disruption to long-term neural and behavioral changes,^30^^,^^31^ the role of biological rhythms in brain development remains less well understood.

The nematode *Caenorhabditis elegans* is a powerful model for investigating how biological rhythms regulate neurodevelopment. During postembryonic development, *C. elegans* progresses through four larval stages, each followed by a molt.^32^ During molting, the worm enters a quiescent state known as developmentally timed sleep (DTS) or lethargus.^33^^,^^34^^,^^35^^,^^36^ Similar to other organisms, DTS is defined by behavioral quiescence, rapid reversibility, increased arousal threshold, stereotypical posture, reduced neuronal activity, and homeostatic regulation.^34^ The timing of molting is regulated by homologs of circadian clock genes,^37^^,^^38^^,^^39^^,^^40^^,^^41^^,^^42^ such as the heterochronic gene *lin-42*, a homolog of the core circadian gene period.^38^^,^^39^^,^^40^ Thus, it has been suggested that components of the circadian clock have been repurposed in *C. elegans* to control molt timing, hereafter referred to as “developmental timing.”^38^^,^^40^

In *C. elegans*, the male nervous system is ∼30% larger than the hermaphrodite’s and undergoes substantial remodeling across postembryonic development.^43^^,^^44^^,^^45^ One key remodeling event is the assembly of the male mating circuitry, which includes 81 male-specific neurons and 89 sex-shared neurons that mediate mating in the adult stage.^46^^,^^47^ Since the connectivity and function of the male mating circuitry are well described, it provides a unique model to study how biological rhythms and DTS influence circuit formation and behavior.

The basic leucine zipper (bZIP) transcription factor (TF) CES-2 has been identified as the *C. elegans* homolog of the *Drosophila* Par domain protein 1ϵ (PDP-1ϵ), which is part of a secondary loop that regulates the fly’s core clock.^41^^,^^42^^,^^48^ It also shares some similarities with the mammalian clock component D-Box binding protein (DBP).^42^ In *C. elegans*, *ces-2* has been shown to regulate apoptosis of one of the NSM sister cells in the embryo and to control the expression of *lin-48* in the excretory duct cell together with another *Drosophila* clock gene homolog, *atf-2*.^49^^,^^50^ However, the role of *ces-2* in postembryonic developmental timing has yet to be explored.

Here, we use *C. elegans* males and their well-defined mating circuitry as a model to investigate the effects of developmental sleep and biological rhythms on synaptic properties and behavior. We first characterize the behavior of males from egg hatching to adulthood and observe that they develop faster than hermaphrodites, with shorter larval stages and DTS episodes. In addition, males spend a smaller fraction of time in quiescence during DTS episodes. We then assess the role of developmental sleep by depriving males of DTS during larval development and find that DTS deprivation does not impair the mating abilities of adult males. Next, we show that *ces-2* affects developmental timing in *C. elegans*; *ces-2* mutant males and hermaphrodites display longer and more variable development times, whereas DTS patterns remain mostly unaffected. Moreover, we demonstrate that *ces-2* males are both less successful in mating and less effective at finding mates. Finally, we show that *ces-2* males present a reduction in the pre-synaptic proteins RAB-3 and CLA-1 within the male mating circuitry. These findings support the notion that homologs of circadian clock genes regulate developmental timing in *C. elegans*, and point to a potential connection between developmental timing and neural circuit formation.

## Results

### Male *C. elegans* exhibit accelerated larval development and reduced sleep

**Figure 1:**
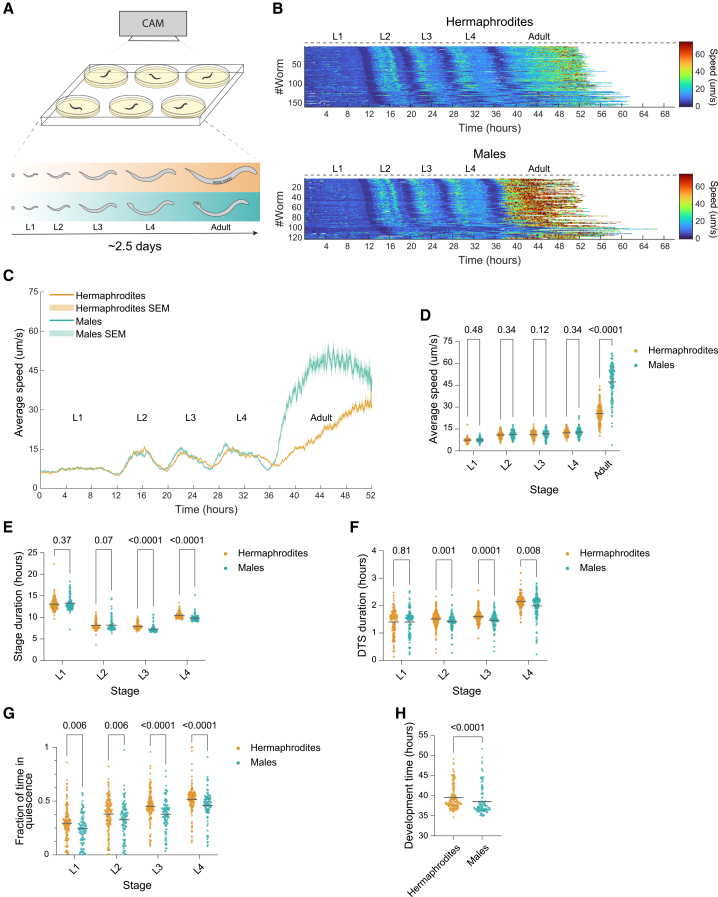
*C. elegans* males exhibit accelerated larval development and reduced sleep (A) Schematic of the imaging system for tracking locomotory behavior of single animals across development. (B) Locomotion speed of hermaphrodites and males from hatching until ∼16 h of adulthood. Each row is a single animal, color represents speed (μm/s). Traces are sorted by entry into the first DTS episode. (Hermaphrodites, *n* = 158; males, *n* = 121.) Animals’ genotype is *him-5(e1490*), used as a wild-type background (see STAR Methods). (C) Average speed across development of hermaphrodites (orange) and males (turquoise). Shaded areas indicate ±SEM. (D) Average speed of hermaphrodites and males in each developmental stage. Statistical significance was evaluated per developmental stage using Mann-Whitney *U* tests with false discovery rate (FDR) correction. (L1 hermaphrodites, *n* = 152; L1 males, *n* = 114; L2–L4 hermaphrodites, *n* = 153; L2–L4 males, *n* = 108; adult hermaphrodites, *n* = 156; adult males, *n* = 119). (E) Duration of each larval stage (i.e., the interval between the end of one DTS episode and the end of the following DTS episode), for hermaphrodites and males. Statistical significance was evaluated using Mann-Whitney *U* tests with FDR correction. (L1 hermaphrodites, *n* = 155; L1 males, *n* = 109; L2–L4 hermaphrodites, *n* = 153; L2–L4 males, *n* = 108). (F) Duration of each developmentally timed sleep (DTS) episode for hermaphrodites and males. Statistical significance was evaluated using Mann-Whitney *U* tests with FDR correction. (L1 hermaphrodites, *n* = 152; L1 males, *n* = 114; L2 hermaphrodites, *n* = 155; L2 males, *n* = 109; L3 hermaphrodites, *n* = 157; L3 males, *n* = 117; L4 hermaphrodites, *n* = 156; L4 males, *n* = 119). (G) Fraction of time spent in quiescence during each DTS episode of hermaphrodites (*n* = 147) and males (*n* = 99). Statistical significance was evaluated using Mann-Whitney *U* tests with FDR correction. (H) Total development time, defined as the amount of time from egg hatching until the exit from the fourth DTS episode, for hermaphrodites (*n* = 156) and males (*n* = 119). Statistical significance was evaluated using Mann-Whitney *U* test. In (B) and (C), data were smoothed using a 10-min moving average. In all figures, each dot represents a single animal; gray bars represent population means, and “*n*” refers to the number of animals.

*C. elegans* transitions between four postembryonic larval stages, which are characterized by cyclic behavioral patterns of active periods and DTS. Although the postembryonic behavioral patterns of hermaphrodites have been well documented,^51^^,^^52^ those of males remain understudied. Sexual dimorphism in circadian rhythms has been observed in mammals and *Drosophila*,^53^^,^^54^^,^^55^^,^^56^ yet it is unclear whether such differences exist in the developmental timing of *C. elegans*. To characterize the behavior of males across development, we tracked individual males and hermaphrodites from egg to adulthood using a long-term imaging system at high spatiotemporal resolution^52^ (Figure 1A, see STAR Methods). For each worm, speed was calculated and used as an indicator of developmental state: wake or DTS (Figures 1B, 1C, and S1A, see STAR Methods). A larval stage was defined as the interval between the end of a DTS episode and the end of the following DTS episode. The speed of males was similar to that of hermaphrodites across all larval stages (L1–L4) and higher at the adult stage (Figure 1D). These findings align with previous studies that found adult-specific sex differences, which coincide with the appearance of pronounced sex differences in body shape.^57^

Interestingly, males progressed through the L3 and L4 stages more rapidly than hermaphrodites and had shorter L2–L4 DTS episodes (Figures 1E and 1F, see STAR Methods). DTS episodes consist of alternating bouts of quiescence and motion.^36^ The fraction of time spent in quiescence within each DTS episode was lower in males across all DTS episodes (Figures 1G and S1B, see STAR Methods). Overall, males exhibited a shorter total development time (Figure 1H). To validate this result, we identified the timing of exit from the L4 molt for individual hermaphrodites and males based on characteristic changes in body size coinciding with molting.^58^^,^^59^ This independent measure also revealed shorter development time in males (Figures S1C and S1D, see STAR Methods). To determine whether the faster development of males was specific to the DTS episodes or reflected a general acceleration of developmental pace, we separately quantified the duration of intermolts: the intervals between DTS episodes. Males exhibited shorter L3 and L4 intermolts than hermaphrodites (Figure S1E), indicating that their faster development is not limited to DTS shortening but extends to the intermolt periods as well. To assess how these differences accumulate over development, we calculated the cumulative stage duration, defined as the sum of all stage durations up to and including the indicated stage (Figure S1F). The cumulative stage durations of males up to L3 and L4 were shorter than those of hermaphrodites. No differences were found in the duration of the L1 and L2 stages and L1 DTS episode, which aligns with previous findings that sex-specific gene expression becomes more pronounced around sexual maturation.^60^ However, differences in the fraction of time spent in quiescence were already evident at L1, suggesting that some sex-specific regulatory mechanisms act earlier in development and affect behavioral states.

### Perturbation of DTS does not impair male mating success

**Figure 2:**
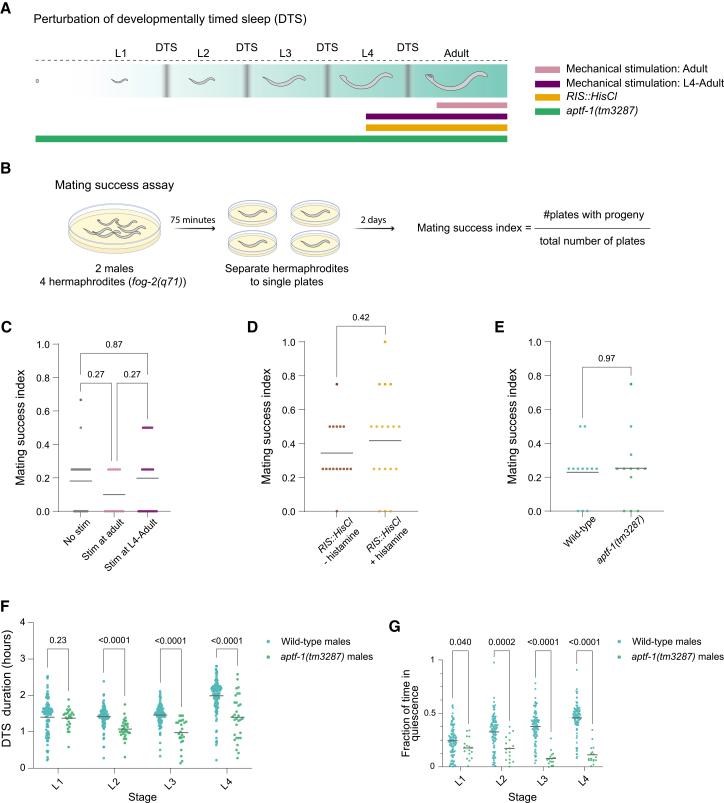
Perturbation of DTS does not affect male mating success (A) Schematic of DTS perturbations. (B) Schematic of mating success assay. (C) Mating success index of wild-type males that were not exposed to mechanical stimulation (gray, *n* = 23), exposed to mechanical stimulation as 1-day adults (pink, *n* = 15) or mechanically stimulated at the L4 to adult transition (purple, *n* = 24). (D) Mating success index of males expressing the *RIS::HisCl* transgene with (brown, *n* = 16) and without (yellow, *n* = 18) histamine application at the L4 to adult transition. (E) Mating success index of wild-type (turquoise, *n* = 12) and *aptf-1(tm3287)* (green, *n* = 12) males. (F) Duration of each DTS episode for wild-type (turquoise) and *aptf-1(tm3287)* males (dark green). (L1 wild-type males, *n* = 114; L1 *aptf-1* males, *n* = 26; L2 wild-type males, *n* = 109; L2 *aptf-1* males, *n* = 33; L3 wild-type males, *n* = 117; L3 *aptf-1* males, *n* = 25; L4 wild-type males, *n* = 119; L4 *aptf-1* males, *n* = 31). (G) Fraction of time spent in quiescence during each DTS episode of wild-type (*n* = 99) and *aptf-1* (*n* = 17) males. All pairwise comparisons were performed using a Mann-Whitney *U* test. FDR correction was applied in (C), (F), and (G) to account for multiple comparisons.

Sleep has been shown to affect neurodevelopment and synaptic plasticity across species,^20^^,^^21^^,^^23^^,^^24^^,^^61^^,^^62^ including *C. elegans*. Therefore, we sought to explore the role of DTS in male mating. DTS perturbation was performed during development (Figure 2A) and followed by a mating success assay; 1-day adult males were placed together with *fog-2* hermaphrodites, which lack sperm and must mate with males to reproduce. Mating success was quantified as the fraction of hermaphrodites that produced progeny, reflecting the proportion of successful mating events that resulted in fertilization (Figure 2B, see STAR Methods). As many of the major remodeling events of the male’s nervous system occur during the L4 to adult transition,^45^^,^^63^^,^^64^ we first tested whether perturbation of the L4 DTS is sufficient to impair mating abilities in adult males. To that end, we either applied mechanical stimulation or silenced the DTS-promoting neuron RIS^65^^,^^66^ using a histamine-gated chloride channel.^67^^,^^68^ We found that neither mechanical stimulation nor RIS silencing during the L4 to adult transition affected adult males’ mating success (Figures 2C and 2D).

To assess the effect of sleep perturbation across development, we tested *aptf-1* mutants, which have been previously shown to sleep less during development.^23^^,^^69^^,^^70^^,^^71^ APTF-1 is a positive regulator of the sleep-inducing neuropeptide FLP-11, which is secreted from the RIS neuron.^72^ Consistent with previous results, *aptf-1* mutants exhibited reduced DTS durations and quiescence in both sexes (Figures 2F, 2G, S1B, and S2A–S2C). Despite having shorter DTS episodes and reduced quiescence fraction, *aptf-1* males showed intact mating success (Figure 2E). Taken together, DTS perturbations by either mechanical stimulation, RIS silencing, or *aptf-1* mutation were not sufficient to affect the mating success of adult males.

### The TF *ces-2* is required for intact developmental timing

In mammals, circadian rhythms have been associated with various neurophysiological processes, including synaptic plasticity, neurodevelopment, and behavior.^30^^,^^31^^,^^73^^,^^74^ Interestingly, although the mammalian molecular clock controls a ∼24-h cycle, some *C. elegans* homologs of circadian clock genes have been shown to influence the timing of molting cycles, which operate on a shorter, developmentally restricted timescale^36^^,^^38^^,^^39^ (Figures 1B and 1E). Given the link between circadian rhythms and neurodevelopment in mammals, we sought to explore whether the formation and function of the male mating circuitry are affected by developmental timing.

**Figure 3:**
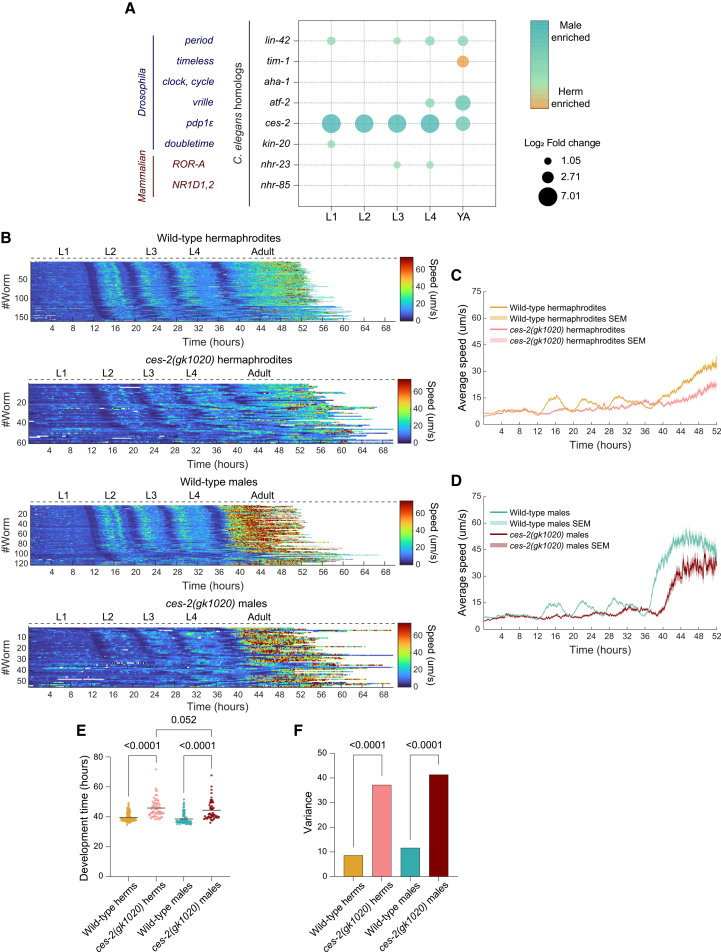
The transcription factor *ces-2* is required for intact developmental timing (A) Sex-specific expression of *C. elegans* clock gene homologs across development. Bubble size represents log_2_ fold change in the expression of that gene between the sexes, and bubble color represents enrichment in either sex. Genes with no bubbles are not significantly enriched in one sex at any time point. Larval stages are denoted as L1–L4; YA, young adult. See also Figure S2 for gene expression profiles. (B) Locomotion speed of wild-type and *ces-2(gk1020)* hermaphrodites (top two images) and wild-type and *ces-2(gk1020)* males (bottom) from hatching until ∼16 h of adulthood. Each row is a single animal; color represents speed (μm/s). Traces are sorted by entry into the first DTS episode. (Wild-type hermaphrodites, *n* = 158; *ces-2* hermaphrodites, *n* = 60; wild-type males, *n* = 121; *ces-2* males, *n* = 55). (C and D) Average speed across development of wild-type (orange) and *ces-2(gk1020)* (pink) hermaphrodites (C) and wild-type (turquoise) and *ces-2(gk1020)* (red) males (D). Shaded areas indicate ±SEM. (E) Total development time of hermaphrodites (*n* = 156), *ces-2(gk1020)* hermaphrodites (*n* = 60), males (*n* = 119), and *ces-2(gk1020)* males (*n* = 54). Statistical significance was evaluated using Mann-Whitney *U* tests. (F) Variance of total development time. Statistical significance was evaluated using *F*-tests for equality of variances. In (B–D), data were smoothed using a 10-min moving average. Traces of wild-type hermaphrodites and males are the same as in Figure 1B.

To that end, we searched for clock-related genes expressed in males during development using the Dimorgena database,^60^ which includes whole-animal, sex-specific gene expression profiles of *C. elegans* across development. Several clock-related genes showed differential expression, with the most prominent being the TF *ces-2*, a homolog of the *Drosophila* clock gene PDP-1ϵ,^41^^,^^42^^,^^48^ which is enriched in males across all developmental stages (Figures 3A and S3). In *C. elegans*, *ces-2* has been shown to regulate apoptosis of one of the NSM sister cells in the embryo and to control the expression of *lin-48* in the excretory duct cell together with another clock gene homolog, *atf-2*.^49^^,^^50^ However, the role of *ces-2* in postembryonic developmental timing has not been explored. To test the effects of *ces-2* on developmental timing, *ces-2* mutants were tracked individually across development (Figures 3B–3D). Interestingly, although most *ces-2* animals displayed the typical four larval stage developmental progression and their associated DTS episodes, they had an obvious alteration in their developmental timing, with a significantly longer and more variable development time (Figures 3E and 3F). A similar developmental delay was also observed in a second *ces-2* allele (*gk892*; Figures S4A–S4C). Although the expression pattern of *ces-2* is male-biased across development, changes in development time were observed in both males and hermaphrodites (Figures 3E and 3F), indicating that *ces-2* plays a role in the hermaphrodite despite its relatively low expression. *ces-2* mutant males displayed shorter L3 and L4 stage durations relative to *ces-2* hermaphrodites (Figure S5A), resembling the sex difference observed in the wild-type (Figure 1E). With respect to DTS, *ces-2* males exhibited shorter L2–L3 DTS episodes, with similar quiescence fraction across all DTS episodes (Figures S5B and S5C). By comparison, wild-type males showed shorter L2–L4 DTS episodes and lower quiescence fraction across all DTS episodes (Figures 1F and 1G). The analysis of intermolt durations further showed that only the L4 intermolt was shorter in *ces-2* males than in *ces-2* hermaphrodites, whereas in wild-type males, both L3 and L4 intermolts were shorter (Figure S1E). Despite these differences, the total development time of *ces-2* males was comparable to that of *ces-2* hermaphrodites (Figure 3E), suggesting that *ces-2* may contribute to, but does not fully account for, the sex differences in developmental timing seen in wild-type animals. In addition, *ces-2* mutant hermaphrodites were slower than wild-types in the L1–L3 stages and in the adult stage, and *ces-2* males were slower across all developmental stages and in the adult stage (Figures S5D and S5E). These findings reveal a previously unrecognized role for *ces-2* in controlling the temporal dynamics of *C. elegans* development.

### *ces-2* mutant males exhibit altered temporal organization of development

**Figure 4:**
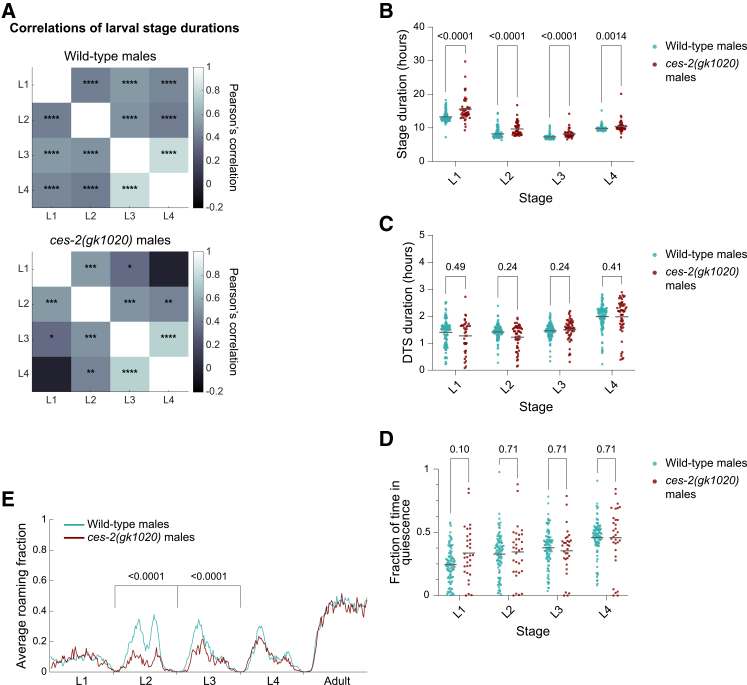
*ces-2* mutant males exhibit altered temporal organization of development (A) Correlation matrices of wild-type (top) and *ces-2(gk1020)* (bottom) males. Color indicates Pearson’s *r* for correlations between every pair of larval stages. Asterisks represent the significance of each Pearson’s correlation, based on two-tailed tests for nonzero correlation. FDR correction was applied to control for multiple comparisons (see STAR Methods). ∗*p* < 0.05; ∗∗*p* < 0.01; ∗∗∗*p* < 0.001; ∗∗∗∗*p* < 0.0001. (B) Duration of each larval stage for wild-type (turquoise) and *ces-2(gk1020)* (red) males. Statistical significance was evaluated using Mann-Whitney *U* tests with FDR correction. (L1 wild-type males, *n* = 109; L1 *ces-2* males, *n* = 50; L2–L4 wild-type males, *n* = 108; L2–L4 *ces-2* males, *n* = 49). (C) Duration of each DTS episode for wild-type and *ces-2(gk1020)* males. Statistical significance was evaluated using Mann-Whitney *U* tests with FDR correction. (L1 wild-type males, *n* = 114; L1 *ces-2* males, *n* = 37; L2 wild-type males, *n* = 109; L2 *ces-2* males, *n* = 50; L3 wild-type males, *n* = 117; L3 *ces-2* males, *n* = 53; L4 wild-type males, *n* = 119; L4 *ces-2* males, *n* = 54). (D) Fraction of time spent in quiescence during each DTS episode of wild-type (*n* = 99) and *ces-2* (*n* = 31) males. Statistical significance was evaluated using Mann-Whitney *U* tests with FDR correction. (E) Average roaming fraction of wild-type (*n* = 121) and *ces-2(gk1020)* (*n* = 55) males across 375 time bins. Significance of the overall difference in each developmental stage was calculated using bootstrapping.

Previous research has shown that despite variability in developmental timing between individuals, the duration of developmental events is proportional to each worm’s development time, a phenomenon defined as temporal scaling.^75^^,^^76^ To test whether temporal scaling is preserved in *ces-2* mutants, Pearson’s correlations were calculated between each pair of larval stage durations for wild-type and *ces-2* mutant males and hermaphrodites (Figures 4A and S5F, see STAR Methods). Wild-type males and hermaphrodites showed significant positive correlations across all stage pairs (Figures 4A and S5F). *ces-2* males lost the L1–L4 correlation (Figure 4A), whereas *ces-2* hermaphrodites lost the L1–L4, L2–L4, and L1–L3 correlations (Figure S5F), indicating disrupted temporal scaling alongside increased variability in development time. In addition, *ces-2* mutants exhibited extended larval stages and intermolt durations (Figures 4B, S1E, and S5G), but their DTS durations were comparable to those of wild-type animals (Figures 4C and S5H), suggesting that *ces-2* regulates the duration of larval stages without affecting DTS duration. The fraction of time spent in quiescence was broadly similar between *ces-2* and wild-type animals across DTS episodes, except for a reduced quiescence fraction in the L3 DTS episode of *ces-2* hermaphrodites (Figures 4D and S5I). Because *ces-2* mutants retained the typical four-stage developmental progression but displayed pronounced alterations in developmental timing and temporal scaling, we asked whether the developmental clock continues to operate in *ces-2* mutants. To address this, we examined the expression dynamics of *lin-42*, the *C. elegans* homolog of the circadian gene *period*, and a well-established marker of the developmental timer.^38^^,^^39^ Consistent with the known oscillatory dynamics of *lin-42*, which peak during each of the larval stages and decay in adults, reporter expression was high in L4 animals and low in adults. This stage-specific pattern was preserved in both wild-type and *ces-2* mutants, in males and hermaphrodites (Figures S6A and S6B, see STAR Methods). These results indicate that the typical difference in expression levels of *lin-42* across the L4 to adult transition persists in *ces-2* mutants, suggesting that CES-2 is not required for the generation of developmental clock oscillations but may instead modulate their timing.

Within each developmental stage, *C. elegans* alternates between two foraging states: roaming and dwelling. Roaming is characterized by a higher speed with a decreased turning rate, and dwelling is characterized by a lower speed with an increased turning rate.^77^^,^^78^ To assess whether *ces-2* mutants present altered behavioral characteristics within developmental stages, each stage was divided into 75 bins, and the fraction of time the worm spent in each of the two foraging states was calculated. *ces-2* males roamed less during the L2 and L3 stages but presented a similar roaming fraction in the L4 and adult stages (Figure 4E). Thus, in addition to timing regulation, *ces-2* also affects behavior within larval stages.

### *ces-2* males exhibit impaired mating and mate-searching behavior, accompanied by altered synaptic organization

**Figure 5:**
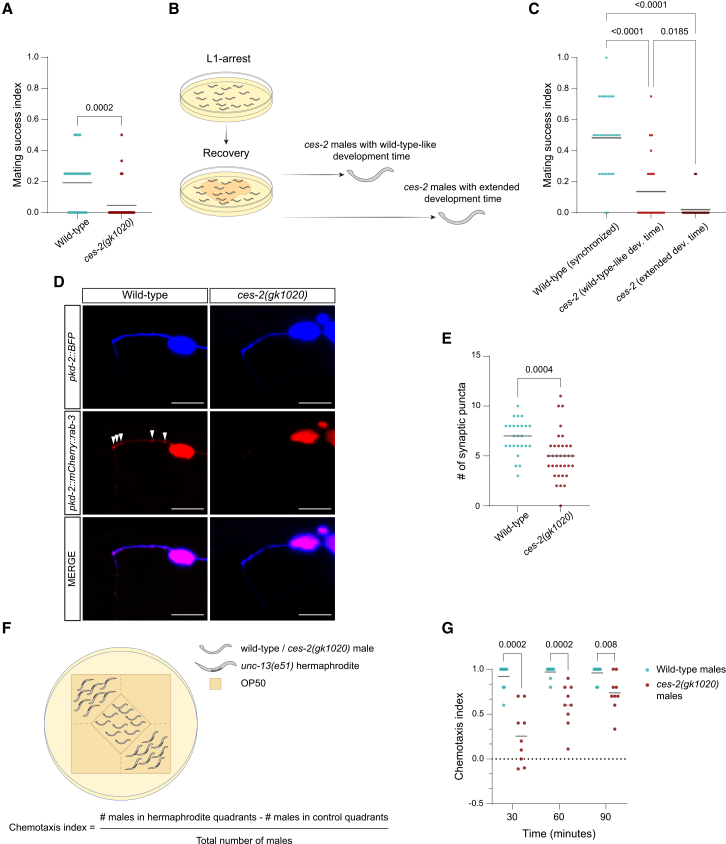
*ces-2* males exhibit impaired mating and mate-searching behavior, accompanied by altered synaptic organization (A) Mating success index of wild-type (*n* = 30) and *ces-2(gk1020)* (*n* = 29) males. Statistical significance was evaluated using Mann-Whitney *U* test. (B) Schematic of synchronization and grouping of *ces-2* males by development time. (C) Mating success index of *ces-2(gk1020)* males with wild-type-like development time and extended development time. Statistical significance was evaluated using Mann-Whitney *U* tests with FDR correction. (Wild-type synchronized males, *n* = 29; *ces-2* [wild-type-like development time], *n* = 23; *ces-2* [extended development time], *n* = 25). (D) Representative confocal micrographs showing *pkd-2::BFP* expression and *pkd-2::mCherry::rab-3* synaptic puncta in the most anterior *pkd-2-*expressing process of 1-day adult wild-type and *ces-2(gk1020)* males. Arrowheads denote synapse location. Scale bars, 10 μm. (E) Number of *pkd-2::mCherry::rab-3* synaptic puncta in wild-type (*n* = 25) and *ces-2(gk1020)* (*n* = 34) males. a.u., arbitrary units. Statistical significance was evaluated using Mann-Whitney *U* test. (F) Schematic of chemo-attraction assay of males toward hermaphrodites. (G) Chemotaxis index of wild-type and *ces-2(gk1020)* males toward mates after 30, 60, and 90 min. Statistical significance was evaluated using Mann-Whitney *U* tests with FDR correction.

Considering the altered developmental timing exhibited by *ces-2* males, we next examined the functionality of their mating circuitry by testing mating success using the same assay described earlier (Figure 2B). *ces-2* males showed a significant decrease in mating success index compared with wild-type males (Figure 5A). As *ces-2* mutants exhibit variability in development time (Figure 3E), the mating defects observed might be attributed to a fraction of the animals with delayed development. To test whether the mating defect depends on overall development time, we subdivided *ces-2* males based on their developmental trajectory using a bleach-synchronized, L1-arrested cohort (Figure 5B, see STAR Methods). *ces-2* males with wild-type-like development time showed higher mating success than *ces-2* males with extended development time, indicating that delayed development is associated with a more severe mating defect. However, both *ces-2* subgroups remained substantially impaired relative to synchronized wild-type males (Figure 5C). These results suggest that although overall development time modulates mating success, additional factors, such as altered temporal scaling, likely contribute to the mating defect observed in *ces-2* mutants.

To test whether the reduced mating success reflected impaired circuit assembly, we quantified the expression of two pre-synaptic proteins, RAB-3 and CLA-1, in a subset of male-specific neurons that are part of the male mating circuitry. To restrict expression to male-specific neurons, we used the *pkd-2* promoter, which drives expression in male-specific ciliated neurons^79^^,^^80^ (Figures 5D and S6C). Although the number of cells expressing the *pkd-2* marker was similar between *ces-2* and wild-type males (Figure S6D), *ces-2* males had a significantly lower expression of CLA-1 in the tail compared with wild-type males (Figures S6C and S6E). Similarly, the number of RAB-3 synaptic puncta along the most anterior neuronal process of the male mating circuitry in the tail was reduced in *ces-2* mutants (Figures 5D and 5E, see STAR Methods). These results indicate that although *ces-2* does not determine the number of male neurons, it is required for their ability to form synapses.

In addition to mating, another function of the male mating circuitry is to facilitate mate-searching behavior, which is mediated by ciliated sensory neurons.^47^^,^^81^^,^^82^ Given the reduced expression of the pre-synaptic markers RAB-3 and CLA-1 in the *pkd-2* ciliated neurons in the tail, we tested the mate-searching behavior of *ces-2* males using a chemo-attraction assay. For that, males were placed at the center of a bacterial lawn, with immobilized hermaphrodites positioned on opposing quadrants. The positions of the males were documented every 30 min for 1.5 h, and a chemotaxis index toward mates was calculated at each time point (Figure 5F, see STAR Methods). We found that *ces-2* males presented a lower chemotaxis index after 30, 60, and 90 min compared with wild-type males (Figure 5G). Overall, these results demonstrate that *ces-2* mutant males are less successful in mating and impaired at mate-searching behavior.

## Discussion

Biological rhythms are thought to affect neurodevelopment, and in many cases, individuals with neurodevelopmental disorders suffer from circadian clock alterations.^14^^,^^17^^,^^18^^,^^19^ However, understanding the underlying mechanisms and providing causal evidence is challenging. Here, we used the well-defined mating circuitry of male *C. elegans* to explore the relationship between biological rhythms and neurodevelopment. We first find that developmental timing is sexually dimorphic in *C. elegans*, with males developing faster than hermaphrodites, emphasizing that sex-specific regulatory mechanisms can influence biological rhythms. We focused on *ces-2*,^41^^,^^42^^,^^60^ the homolog of the *Drosophila* clock gene PDP-1ε, which is male-enriched across all larval stages. We discovered that *ces-2* mutant males and hermaphrodites exhibit defective developmental timing, characterized by elongated larval stages, increased inter-individual variability in development time, and altered temporal scaling, without apparent effects on DTS duration or quiescence fraction. Notably, despite these timing defects, *ces-*2 mutants retained the canonical four larval stage developmental progression and the typical difference in expression levels of the core clock marker *lin-42*, with high expression in L4 and reduced expression in adults. Thus, the core developmental clock remains active in *ces-2* mutants, whereas its temporal regulation is disrupted. These findings are in line with previous studies demonstrating that homologs of circadian clock genes affect the timing of developmental events in *C. elegans*,^39^^,^^40^ suggesting that the same molecular components have been adapted to control two distinct timing systems: circadian rhythms and developmental timing.

Previous studies on clock gene homologs that regulate developmental timing in *C. elegans* have mainly focused on larval development rather than adult behavior and neuronal circuit formation. As the male’s mating circuitry undergoes major remodeling events across development, we used it as a model to explore the link between developmental timing and neurodevelopment. To that end, we tested the mating abilities of adult males and discovered that *ces-2* mutant males had reduced mating success compared with wild-types, with more severe defects in animals with delayed development. However, even *ces-2* males with wild-type-comparable development time remained less successful than wild-type males. This result indicates that delayed development contributes to the mating defect, while suggesting that additional timing-related disruptions, such as impaired temporal scaling, as well as potential timing-independent effects, may further affect circuit function. These findings are consistent with the hypothesis that biological rhythms are important regulators of neurodevelopment and adult behavior. To determine whether the observed defects reflect changes in the male mating circuitry, we quantified the extent of connectivity formed by male-specific neurons within the circuit. We found that in *ces-2* males, male-specific neurons within the male mating circuitry formed fewer synapses, which may indicate abnormal neuronal remodeling events in *ces-2* mutants. Because ciliated sensory neurons in the male mating circuitry are thought to regulate mate-searching behavior,^47^^,^^81^^,^^82^ we examined this behavior using a chemo-attraction assay, and found that *ces-2* males exhibit impaired mate-searching behavior. Although *ces-*2 males are slower than wild-type males at the adult stage, the scale and duration of the assay make it unlikely that reduced locomotion alone limits access to the hermaphrodites.

Developmental timing in *C. elegans* has classically been studied through heterochronic genes that regulate stage-specific cell fates.^83^ Subsequent work linked this system to circadian clock components, including the *lin-42*/PERIOD homolog, which controls the timing of larval transitions.^38^^,^^39^ Our findings extend this framework by suggesting that, beyond specifying temporal identity, the coordination and scaling of developmental events are important for proper neuronal circuit assembly and function.

Together, our results suggest that the precise timing of developmental events is crucial for the proper assembly of the male mating circuitry in *C. elegans*. As *ces-2* also regulates apoptosis and transcription in other contexts,^49^^,^^50^ its effects on neuronal circuit formation may not be exclusively mediated through developmental timing, and further work will be required to distinguish between these roles.

One of the most prominent manifestations of biological rhythms is the sleep-wake cycle.^4^ The importance of sleep for neurodevelopment has been well established.^20^^,^^21^^,^^22^^,^^23^^,^^24^^,^^25^^,^^27^^,^^61^ Here, we first examined developmental sleep patterns in males and found that they exhibit shorter L3 and L4 DTS episodes, with a smaller quiescence fraction across all DTS episodes, compared with hermaphrodites. Considering the strong association between sleep and synaptic plasticity, and the remodeling events that occur in the male’s nervous system across postembryonic development, we hypothesized that DTS plays a role in the formation of the male mating circuitry. To test that, we used three different methods to impair DTS, either across development or during the L4 to adult transition, when many neurodevelopmental events occur. Surprisingly, we found that perturbations of DTS by mechanical stimulation, genetic mutation, or silencing of the sleep-promoting neuron RIS were not sufficient to affect the mating success of adult males. This may reflect the presence of compensatory mechanisms that preserve mating ability in males, who must mate to reproduce. Additionally, sleep disruption may affect circuit properties in ways that are not captured by the mating success assay, as previous research demonstrated that sleep perturbation affects the properties of the DVB neuron in males.^23^

Overall, our results demonstrate that developmental timing in *C. elegans* is sexually dimorphic and suggest that precise timing is crucial for proper assembly of the male mating circuitry. The circadian clock gene homolog *ces-2* appears to play a key role in this process, as its disruption results in delayed and desynchronized development, reduced synaptic connectivity, and impaired mating ability and mate-searching behavior. Although DTS deprivation did not impair mating success, our findings support a model in which precise developmental timing, regulated by clock gene homologs, is essential for proper circuit formation and behavior. The conserved role of molecular clock components in regulating the developmental timing of *C. elegans*, together with the many advantages of this model organism, highlight its potential as a powerful platform for studying the relationship between sex, biological clocks, and neurodevelopment.

### Limitations of the study

Several limitations should be considered when interpreting our findings. Although our results support a link between altered developmental timing and defects in neuronal circuit formation and behavior, they do not establish direct causality. Because *ces-2* has known roles in apoptosis and transcriptional regulation, we cannot completely exclude the possibility that its effects on circuit formation are partially independent of its role in developmental timing. Although we observed broadly consistent developmental phenotypes across *ces-2* alleles, some differences in variability may reflect allele-specific effects or differences in sample size. It should be noted that both *ces-2(gk1020)* and *ces-2(gk892)* mutants were backcrossed twice. Furthermore, although we used multiple approaches to perturb developmental sleep, we did not directly validate the effectiveness of all sleep disruption methods due to technical constraints. Although these approaches have been previously shown to reduce sleep in several studies, we were unable to monitor sleep during perturbation in our experimental setup. Finally, our assessment of synaptic connectivity is based on structural measurements and does not directly address functional synaptic activity.

## Resource availability

### Lead contact

Further information and requests for resources and reagents should be directed to and will be fulfilled by the lead contact, Meital Oren-Suissa (meital.oren@weizmann.ac.il).

### Materials availability

Unique strains generated in this study have been deposited at the Caenorhabditis Genetics Center. Requests for other strains and plasmids should be directed to the lead contact.

### Data and code availability

• All tracking data generated in this study have been deposited at the Weizmann Institute of Science Research Data repository: https://doi.org/10.34933/a4ed89c9-a3ee-47e9-a8c3-415f6e484a0f and are publicly available as of the date of publication. Any additional data reported in this paper will be shared by the lead contact upon request.
• All original code has been deposited at the Weizmann Institute of Science Research Data repository: https://doi.org/10.34933/a4ed89c9-a3ee-47e9-a8c3-415f6e484a0f. Code for extraction of behavioral trajectories is available at GitHub: https://github.com/ChristophKirst/CelegansLongTermBehavioralAnalysis/tree/master/matlab (Stern et al.^52^).
• Any additional information required to reanalyze the data reported in this paper is available from the lead contact upon request.

## Acknowledgments

We thank members of the Oren-Suissa laboratory for their critical insights regarding the manuscript. Special thanks to Dr. Yehuda Salzberg and Dr. Rizwanul Haque for their assistance and valuable conceptual input and to Dolev Galski for his assistance with strain generation. We thank Scott Emmons and Michael P. Hart for their generosity in sharing strains and Anthony D. Fouad from Tau Scientific for technical support with the WormWatcher vibration stage. Some strains used in this study were obtained from Caenorhabditis Genetics Center (CGC), which is funded by the NIH 10.13039/100016958Office of Research Infrastructure Programs (P40 OD010440). *ces-2(gk1020)* and *ces-2(gk892)* were provided by the *C. elegans* Reverse Genetics Core Facility at the University of British Columbia. We thank WormAtlas, which is supported by the NIH OD 010943. We thank WormBase, an online biological database for *C. elegans*, which is supported by Grant U41 HG002223 from the 10.13039/100000051National Human Genome Research Institute at the NIH, the 10.13039/501100000265UK Medical Research Council, and the UK Biotechnology and Biological Sciences Research Council. 10.13039/100003941M.O.-S. acknowledges financial support from the European Research Council ERC-2019-STG 850784, European Research Council ERC-2024-COG 101169837, Israel Science Foundation grant 961/21, 10.13039/100010661Horizon TMA MSCA Doctoral Networks grant # 101119745, Dr. Barry Sherman Institute for Medicinal Chemistry, Sagol Weizmann-MIT Bridge Program, and the 10.13039/100021131Azrieli Foundation. S.S. acknowledges financial support from the European Research Council ERC-STG 851634 and Israel Science Foundation grant 3035/20.

## Author contributions

S.N.H. performed all experiments except long-term tracking experiments and carried out the data analysis. E.N. conducted tracking experiments and initial data processing and performed the analyses of roaming fraction, body size, and quiescence fraction. S.S. and M.O.-S. guided and supervised the work. S.N.H. and M.O.-S. wrote the paper.

## Declaration of interests

The authors declare no competing interests.

## Declaration of generative AI and AI-assisted technologies in the writing process

During the preparation of this work, the authors used ChatGPT in order to check for grammar, and for code optimization. After using this tool, the authors reviewed and edited the content as needed and take full responsibility for the content of the publication.

## STAR★Methods

### Key resources table

**Table:** 

REAGENT or RESOURCE	SOURCE	IDENTIFIER
**Bacterial and virus strains**		

*E. coli*: OP50	Caenorhabditis Genetics Center	N/A

**Deposited data**		

Original code, figure data, and raw data	Weizmann Institute of Science CRIS (Current Research Information System)	Weizmann Institute of Science CRIS (Current Research Information System): https://doi.org/10.34933/a4ed89c9-a3ee-47e9-a8c3-415f6e484a0f

**Experimental models: Organisms/strains**		

Wild-type: *him-5(e1490)V*	CGC	CB4088
*him-5(e1490) V; aptf-1(tm3287) II*	This study	MOS843
*ces-2(gk1020) I; him-5(e1490) V*	This study	MOS737
*ces-2(gk892) I; him-5(e1490) V*	This study	MOS1017
*him-8(e1489) IV; otIs525[lim-6*^*int4*^*::gfp]; qnEx643[flp-11p::HisCl::SL2::mCherry, myo-2p::mCherry]*	Cowen et al.^23^	MPH41
*him-5(e1490) V; qnEx643[flp-11p::HisCl::SL2::mCherry, myo-2p::mCherry]*	This study	MOS884
*bxls33[pkd-2p::3xnovoGFP*::*cla-1, pkd-2p::mCherry::rab-3, pkd-2p::BFP] I; him-5(e1490) V*	Sakai et al.^79^	EM1890
*ces-2(gk1020) I; him-5(e1490) V; bxIs33[pkd-2p::3xnovoGFP*::*cla-1, pkd-2p::mCherry::rab-3, pkd-2p::BFP] I*	This study	MOS931
*bafIs62 [lin-42p::GFP +unc-119(+)]; him-5(e1490) V*	This study	MOS1020
*bafIs62 [lin-42p::GFP +unc-119(+)]; ces-2(gk1020); him-5(e1490) V*	This study	MOS1030

**Software and algorithms**		

MATLAB	MathWorks	https://www.mathworks.com/products/matlab.html
GraphPad Prism	GraphPad	https://www.graphpad.com
ImageJ	National Institutes of Health (NIH)	https://imagej.net/ij/
Adobe Illustrator	Adobe	https://www.adobe.com
FlyCapture	FLIR	https://www.flir.com/
ZEN	ZEISS	https://www.zeiss.com/microscopy/en/products/software/zeiss-zen.html

**Other**		

12 MP USB3 Flea camera	FLIR	Cat#FL3-U3-120S3C-C
LED backlights	Metaphase Technologies	Cat#99021169
Temperature control (cooling unit- Peltier element)	TE technology	Cat#AC-027
Code for extraction of behavioral trajectories	Stern et al.^52^	https://github.com/ChristophKirst/CelegansLongTermBehavioralAnalysis/tree/master/matlab

### Experimental model and study participant details

#### *C. elegans* strains

Worms were maintained according to standard methods.^84^ *C. elegans him-5(e1490)* were used as wild-type controls for all strains with this allele in their background. Both sexes (males and hermaphrodites) were examined across all four larval stages (L1–L4) and the adult stage. The developmental stage and sex of the animals are specified for each experiment. Worms were grown on nematode growth media (NGM) plates seeded with bacteria (*E. coli* OP50) as a food source, at 22.5°C for behavioral tracking and at 20°C for all other assays. For long-term behavioral tracking, worms were bleached to isolate embryos. Single embryos were then transferred into custom-designed laser-cut multi-well plates where the size of each well is 10 mm in diameter. Each well contained 10 μL of *E. coli* OP50 (OD 1.5) that was UV killed, to prevent bacterial growth during the experiment.

#### Sex as a biological variable

This study examined both males and hermaphrodites. Sex was treated as a biological variable, and sex differences in developmental timing and sleep patterns are reported throughout.

### Method details

#### Imaging system

Behavioral monitoring was performed by a longitudinal imaging system that consists of an array of six 12 MP USB3 cameras (Flea3, FLIR), each with 35 mm high-resolution objectives (Edmund Optics) and mounted on optical construction rails (Thorlabs). Each camera captures one experimental plate containing up to six wells, allowing for simultaneous imaging of 36 isolated individuals across development. Movies are recorded at 3 frames per second with a spatial resolution of ∼9.5 μm. The array of cameras was enclosed within environmentally controlled chamber in which temperature is controlled using a Peltier element (TE Technologies) and maintained at 22.5 ± 0.07 °C, and humidity is held in the range of 50 ± 5% with a sterile water reservoir. To maintain uniform illumination, LED backlights (Metaphase Technologies) with polarization sheets served as the only light source. Each camera continuously captured up to 300–400 short movies (∼12 min each) under standardized settings: Gain = 0, Exposure = 1.17, Shutter = 10.964, White Balance (Red) = 711, and White Balance (Blue) = 563. Movies from the cameras were captured using commercial software (FlyCapture, Pointgrey).

#### Behavioral trajectory extraction from imaging data

To monitor the behavior of individuals across their entire developmental trajectories, recorded videos were analyzed using custom MATLAB scripts (MathWorks, version 2021b) as previously described (Stern et al.^52^). Worms were detected as moving objects via background subtraction, and their center-of-mass coordinates were extracted for each frame within each behavioral arena. Egg hatching time of each individual in the experiment is automatically marked by the time when activity can be detected in the behavioral arena. The middle of the lethargus periods, in which animals stop their locomotion and molt, were defined as the transition points between different stages of development (based on 10 s timescale speed trajectories over time, smoothed over 300 frames). Locomotory speed was calculated over a 1-s time window. Quantification of roaming and dwelling behavior across development: Roaming states are defined by high speed and low angular velocity, while dwelling states are defined by low speed and high angular velocity episodes (Ben Arous et al.^78^; Flavell et al.^77^; Stern et al.^52^). Speed and angular velocity were calculated over a 10-s rolling window, and a 2D probability map (50 × 50 bins) was generated per time bin (speed bin: 7.59 μm/s; angular velocity bin: 3.6°/s) (Stern et al.^52^). For each development stage, a threshold slope was defined (5, 2.5, 2.3, 2, and 1.5 for the L1-L4 larval stages and adult stage, respectively) to classify roaming and dwelling states in each frame. The number of frames that each individual spent in the roaming state within a given time bin was then calculated, representing its roaming fraction.

#### DTS automatic analysis

DTS automatic analysis was performed using a custom-made script programmed in MATLAB (Mathworks, version 2023b) which was based on the lethargus analysis from Harel et al.^85^ DTS episodes were automatically identified for each worm around the manually annotated developmental stage boundaries based on the worm’s speed. For each of the four larval transitions, a search window was defined around the manually annotated stage boundary. The window started 70% of the way from the previous boundary to the current one, and ended 70% of the way from the current boundary to the next one. Within each window, the speed data was segmented into non-overlapping bins of 300 frames each, and the mean speed of each bin was used to compute an empirical cumulative distribution function (ECDF). The ECDF curve was smoothed using a Gaussian filter with a window size of 10 bins, and missing values were replaced with a default of 1. DTS onset and offset were defined as the points surrounding the molt where the smoothed ECDF dropped below and rose above a behavioral threshold of 0.2. Only DTS episodes that were successfully identified by the automatic analysis were used for further analysis. Detection rate was calculated as the percentage of DTS episodes successfully identified out of the total expected number of DTS episodes (Figure S1A). Since each animal undergoes four DTS episodes, the expected total was defined as 4 × number of animals.

#### Developmental timing analysis

All intervals were calculated per individual worm based on its DTS automatic analysis. Stage duration was defined as the interval from the end of a DTS (or from the start of the recording for L1) to the end of the following DTS. Intermolt duration was defined as the interval from the end of a DTS (or from the start of the recording for L1) to the start of the following DTS. Development time was defined as the time between the start of the recording and the exit from the fourth DTS episode. Cumulative stage duration was calculated as the sum of all stage durations up to and including the stage of interest. For example, the cumulative duration at L2 corresponds to L1 + L2, at L3 to L1 + L2 + L3, and at L4 to L1 + L2 + L3 + L4.

#### Quiescence fraction analysis

Quiescence during DTS episodes was quantified using the animal’s displacement of the center-of-mass, measured over 10-s intervals. These displacements were continuously quantified based on the existing behavioral tracking data. For each individual, the quiescence fraction within each of its four defined DTS windows (All transitions across L1-Adulthood) was calculated as the number of 10 s windows in which displacement value was below a threshold of 30 μm, divided by the total number of windows within that DTS window. This threshold was determined based on identified periods of minimal movement across individuals. Only worms for which all four DTS episodes were successfully detected by the automated DTS analysis were included in this analysis.

#### Detection of developmental transitions by worm size measurements

The size of each individual across development was quantified using frame-by-frame pixel data generated from the tracking system. For each individual, image processing extracted the total number of pixels in every frame, which indicates the worm’s size. To construct a continuous developmental trajectory for each individual, size traces from multiple movies of the same individual were concatenated into a single continues trajectory of the individual’s growth across the experiment. To minimize high-frequency noise, the resulting trajectories were smoothed using a moving window of 30 frames. These smoothed growth trajectories were then used to detect stage transitions across development of each individual by manually identifying transient drops in measured body size, which are known to correspond to molting period, during which worms enter lethargus.^58^^,^^59^ In particular, we detected the L4 to adult transition by identifying the point at which the smoothed size trajectory begins to increase again following the clear size drop that occur after approximately 28 h of development (∼300,000 frames at 3 frames per second), to quantify the overall time of larval development in both hermaphrodites and males. Importantly, the threshold used to detect the worm in the image changes at movie 100 such that during early development, a lower threshold is used to ensure detection of small worms, whereas after movie 100, when worms are larger, a higher threshold is applied to reduce noise. As a result, an apparent drop in measured size is often observed at this point, reflecting a technical change rather than a biological effect.

#### Histamine-mediated RIS silencing

NGM-Histamine plates were prepared as described: 10 mM NGM-histamine was prepared by adding 5 mL of 1M histamine dihydrochloride to 500 mL of the agar while stirring. NGM histamine and control agar (with no addition of histamine) were then poured into labeled petri dishes. NGM-histamine (10 mM) and control plates were stored at 4°C for no longer than 2 months. Histamine plates were tested using worms that carry a transgene with a pan-neuronal HisCl1 (*tag168::HisCl1::SL2::GFP*).^67^ After a few minutes on histamine plates, these worms were paralyzed completely, validating the potency of the histamine plates. Transgenic worms expressing a histamine gated chloride channel in RIS (*flp-11p::HisCl::SL2::mCherry*) were used to apply a histamine-mediated DTS perturbation by RIS silencing. For perturbation of the L4 DTS, L4 transgenic males were transferred to NGM histamine or control plates with a thin lawn of OP50 and stored overnight at 20°C until the assay the following morning. The males were left on the histamine plates until the mating success assay to avoid compensatory sleep.

#### Mechanical stimulation

Early to mid-L4 *him-5(e1490)* males or 1-day adult controls were picked into fresh NGM plates containing a lawn of OP50 a day before the experiment and placed on a WormWatcher vibration stage made by Tau Scientific. A 12,000 Hz vibration pulse was applied for 1 s every 10 s across the L4 to adult transition. The males were left on the vibration stage until the mating success assay to avoid compensatory sleep.

#### Mating success assay

6 cm NGM assay plates were seeded with 10 μL OP50 two days before the experiment and kept at 20°C. One day before the experiment, L4 males and *fog-2(q71)* hermaphrodites were isolated and maintained at 20°C overnight. On the day of the experiment, four 1-day adult *fog-2(q71)* hermaphrodites were placed on the bacterial lawn within an assay plate with two 1-day adult males and left to mate for 75 min. After 75 min, each *fog-2(q71)* hermaphrodite was transferred to a fresh NGM plate containing a lawn of OP50 and stored at 20°C for two days. Two days later, the mating success index of the males was calculated by counting the number of plates that contained progeny out of the total number of plates (Figure 2B). The quantification was performed separately for each pair of males that were placed on the same assay plate.

#### Synchronization and grouping of *ces-2* males by development time

Gravid *him-5(e1490)* and *ces-2(gk1020)* adults were bleached to obtain synchronized embryos, which were incubated overnight on unseeded NGM plates to induce L1 arrest. The following morning, L1-arrested larvae were washed in M9, transferred onto seeded plates, and allowed to develop at 20°C. One day later, L4 *him-5* males and *ces-2* males with wild-type-like development time (i.e., reaching L4 on the same day as *him-5*) were picked and incubated overnight at 20°C. The next day, L4 *ces-2* males with extended development time (reaching L4 one day later) were picked from the same plates and incubated under the same conditions. All males were assayed for mating success as day 1 adults.

#### Chemo-attraction assay

To test the ability of males to locate potential mates we modified the quadrant assay from.^86^ *him-5(e1490)* and *ces-2(gk1020)* males, and *unc-13(e51)* hermaphrodites were isolated one day before the experiment and stored at 20°C overnight. A 3 × 3 cm square divided into four quadrants was marked on the bottom of 9 cm NGM assay plates. A 0.5 × 0.5 rhombus was marked in the middle of the square. 50 μL OP50 were seeded and spread across the 3 × 3 cm square using a fine spreader one day before the experiment. On the day of the assay, eight 1-day adult *unc-13(e51)* hermaphrodites were placed on opposite quadrants of the square (hermaphrodite quadrants) and left for 30 min at room temperature, the other two quadrants served as controls (control quadrants). Next, ten *him-5* or *ces-2* males were placed within the 0.5 × 0.5 rhombus and left to roam for 90 min (Figure 4HA). The position of the males was documented after 30, 60 and 90 min. Chemotaxis index was calculated as: CI = (# males in hermaphrodite quadrants - # males in control quadrants)/(Total number of males).

#### Microscopy

Animals were mounted on a 5% agarose pad on a glass slide, on a drop of M9 containing 100–200 mM sodium azide (NaN3) as an anesthetic. A Zeiss LSM 880 confocal microscope was used with 40× magnification. Zen software was used for imaging (version 2.3).

#### Quantification of synapses in the tail

To assess the amount of synapses within the male mating circuitry, which is mostly located in the tail, 1-day adult transgenic males expressing *pkd-2::mCherry::rab-3* and *pkd-2::3xnovoGFP::cla-1* to mark synapses, and *pkd-2::BFP* to identify the *pkd-*2-expressing cells were used. For every worm, the same rectangle-shaped ROI (349 × 596 pixels) was placed around the male-specific neurons in the tail, which were identified using *BFP* expression. Fluorescence intensity of the *3xnovoGFP* signal was quantified in maximum intensity projection using ImageJ version 1.54p. To correct for background fluorescence, a second rectangular ROI (65 × 50 pixels) was placed adjacent to the *3xnovoGFP*-positive region in an area lacking signal. The mean intensity of this background ROI was subtracted from the mean intensity of the ROI containing the signal. This procedure was applied consistently across all images. The number of *RAB-3* puncta was quantified in the most anterior *pkd-2-*expressing process which extends from the *pkd-2*-expressing neuron located farthest from the tail tip. This area was selected because it is easy to identify and allows high-resolution visualization of discrete synaptic puncta despite a relatively noisy *pkd-2p::mCherry::rab-3* signal. Only animals in which this anterior process could be clearly identified were included in the analysis. One data point was excluded from the *3xnovoGFP* analysis based on outlier detection using the “Identify Outliers” function in GraphPad Prism (ROUT method, Q = 1%).

#### *lin-42::GFP* expression analysis

To assess developmental clock activity, *lin-42::GFP* reporter expression was analyzed in L4 larvae and 1-day-old adults. For each animal, *lin-42::GFP* expression was scored qualitatively as either high or low based on fluorescence intensity in the pharyngeal region. Classification criteria were defined prior to analysis and applied consistently across all samples. Animals with visible fluorescence in both pharyngeal bulbs were classified as high expression, whereas animals with weak or undetectable fluorescence were classified as low expression. For each condition, the percentage of animals in each category was calculated.

### Quantification and statistical analysis

Pairwise comparisons of genotype or sex at each developmental stage (L1–L4, and Adult where applicable), as well as chemotaxis index, were performed using two-sided Mann–Whitney U tests in MATLAB R2023b. For multiple stages or time-points parameters (e.g., larval stage durations, DTS durations, average speed, and chemotaxis index), *p*-values were adjusted using the Benjamini-Hochberg false discovery rate (FDR) correction. For single timepoint measures (e.g., total development time), uncorrected *p*-values are reported. The statistical significance of the differences in average roaming behavior between strains was calculated by bootstrapping. In each developmental stage, the observed differences in average roaming fraction was compared to a null distribution of differences in average roaming activity, generated by randomly resampling the combined dataset 1000 times. For speed data, outlier data points were removed by calculating the mean and standard deviation for each worm during L1–L4, and setting values exceeding the mean by more than 3 standard deviations to NaN. For visualization, the data was smoothed using a 10-min moving average (1800 frames at 3 Hz).

#### Temporal scaling analysis

To assess the relationships between larval stage durations, Pearson’s correlation coefficients and their associated two-tailed *p* values for nonzero correlation were computed using MATLAB’s built-in “corr” function with pairwise deletion for handling missing data. Correlation matrices were computed separately for each sex and genotype, evaluating pairwise correlations between the four larval stages within each group. A Benjamini-Hochberg FDR correction was applied to control for multiple comparisons.

All other statistical analyses were performed using GraphPad Prism 10. Each figure or figure legend includes information regarding statistical testing and sample size.
