# Supplementary material for: A circadian clock gene homolog regulates developmental timing and male mating circuitry in C. elegans
Source: iScience. 2026 Jul 2;29(7):116659. doi: 10.1016/j.isci.2026.116659 (PMC13355510; doi:10.1016/j.isci.2026.116659)
Supplement: Document S1. Figures S1–S6 [file mmc1.pdf]

iScience, Volume *29*

Supplemental information

A circadian clock gene homolog regulates developmental timing and male mating circuitry in *C. elegans*

Shiraz Nir Halber, Eshkar Nir, Shay Stern, and Meital Oren-Suissa

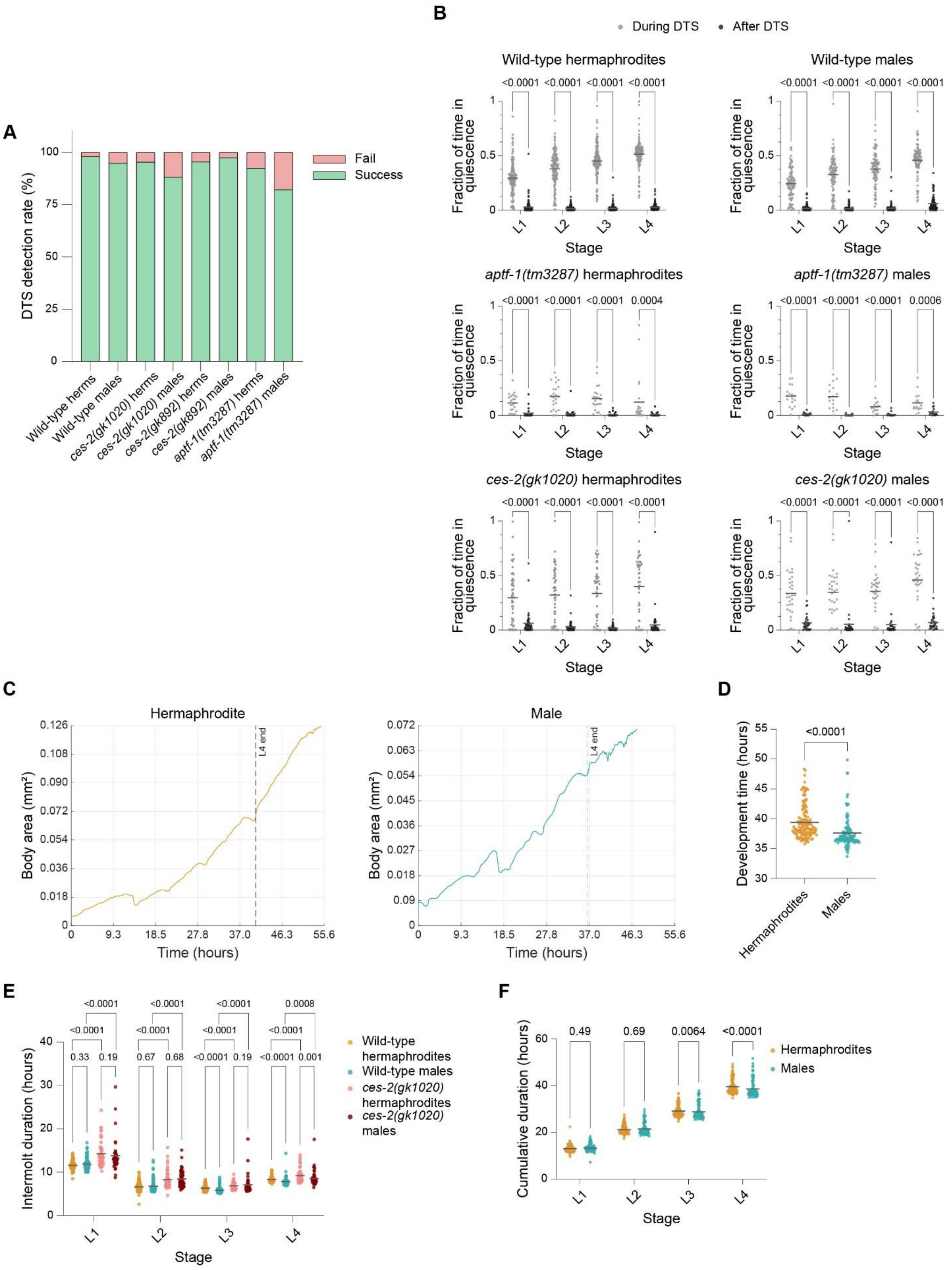
A
DTS detection rate (%)
100
75
50
25
0
Fail
Success
Wild-type herms
Wild-type males
ces-2(gk1020) herms
ces-2(gk1020) males
ces-2(gk892) herms
ces-2(gk892) males
aptf-1(tm3287) herms
aptf-1(tm3287) males
B
During DTS
After DTS
Wild-type hermaphrodites
Wild-type males
aptf-1(tm3287) hermaphrodites
aptf-1(tm3287) males
ces-2(gk1020) hermaphrodites
ces-2(gk1020) males
<0.0001
0.0004
0.0006
Fraction of time in quiescence
Stage
L1
L2
L3
L4
C
Hermaphrodite
Male
Body area (mm²)
Time (hours)
L4 end
0.126
0.108
0.090
0.072
0.054
0.036
0.018
0.072
0.063
0.054
0.045
0.036
0.027
0.018
0.09
0
9.3
18.5
27.8
37.0
46.3
55.6
D
Development time (hours)
<0.0001
55
50
45
40
35
30
Hermaphrodites
Males
E
Intermolt duration (hours)
<0.0001
0.33
0.19
0.67
0.68
0.19
0.0008
0.001
40
30
20
10
0
Wild-type hermaphrodites
Wild-type males
ces-2(gk1020) hermaphrodites
ces-2(gk1020) males
Stage
F
Cumulative duration (hours)
0.49
0.69
0.0064
<0.0001
60
40
20
0
Hermaphrodites
Males
Stage

Figure S1. DTS- and size-based analyses of developmental timing, related to Figures 1,2 and 4
(A) Detection rate of the DTS automatic analysis for each genotype.
(B) Fraction of time spent in quiescence during DTS episodes (grey) and during a time window of equal duration shifted 30,000 frames (3fps) later (black) for wild-type hermaphrodites (n = 147), wild-type males (n = 99), *aptf-1* hermaphrodites (n = 23), *aptf-1* males (n = 17), *ces-2* hermaphrodites (n = 50), *ces-2* males (n = 31). Statistical significance was evaluated using Mann-Whitney U tests with FDR correction.
(C) Representative body size trajectories for a hermaphrodite (left) and a male (right) across development. The dashed line indicates the time point of exit from the L4 molt, identified as the onset of renewed growth following the characteristic transient decrease in body size. An apparent drop in measured size is often observed around movie 100 due to a technical change in the detection threshold and is unrelated to molting (see *Methods*).
(D) Total development time, defined as the amount of time from egg hatching until the exit from the fourth molt based on body size analysis, for hermaphrodites (n = 149) and males (n = 109). Statistical significance was evaluated using Mann-Whitney U test.
(E) Duration of each intermolt for wild-type and *ces-2* hermaphrodites and males. Statistical significance was evaluated using Mann-Whitney U tests with FDR correction. (L1 wild-type hermaphrodites, n = 152; L1 wild-type males, n = 114; L1 *ces-2* hermaphrodites, n = 53; L1 *ces-2* males, n = 37; L2 wild-type hermaphrodites, n = 154; L2 wild-type males, n = 105; L2 *ces-2* hermaphrodites, n = 56; L2 *ces-2* males, n = 49; L3 wild-type hermaphrodites, n = 150; L3 wild-type males, n = 112; L3 *ces-2* hermaphrodites, n = 53; L3 *ces-2* males, n = 36; L4 wild-type hermaphrodites, n = 154; L4 wild-type males, n = 105; L4 *ces-2* hermaphrodites, n = 56; L4 *ces-2* males, n = 49).
(F) Cumulative duration of developmental stages for wild-type hermaphrodites and males, plotted as the sum of stage durations up to and including the indicated stage. Statistical significance was evaluated using Mann-Whitney U tests with FDR correction. (L1 hermaphrodites, n = 155; L1 males, n = 109; L2–L4 hermaphrodites, n = 153; L2–L4 males, n = 108).
Each dot represents a single animal; grey bars represent population means.

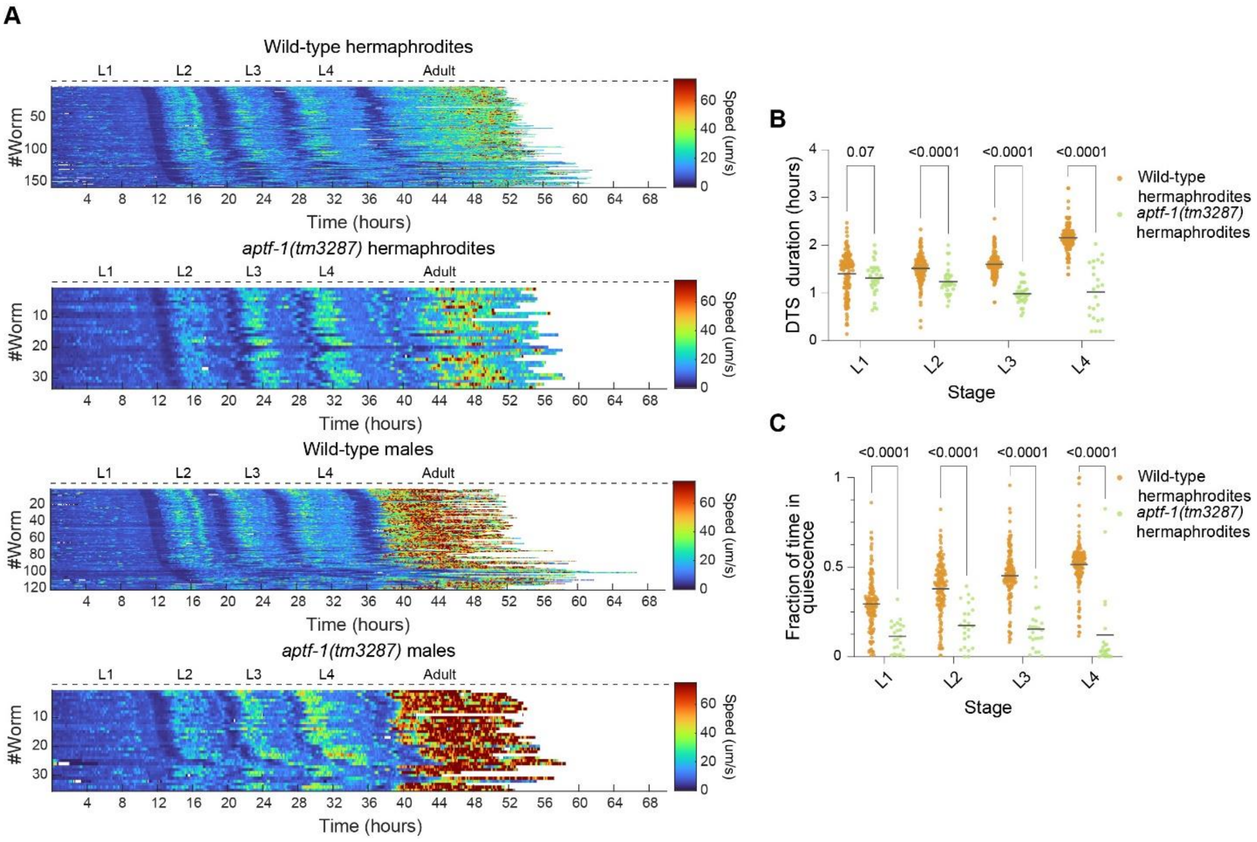

Figure S2. ***aptf-1*** **mutant data, related to Figure 2**

(A) Locomotion speed of wild-type and *aptf-1(tm3287)* hermaphrodites (top two panels) and wild-type and *aptf-1(tm3287)* males (bottom panels) from hatching until ~16 hours of adulthood. Traces are sorted by entry into the first molt. (wild-type hermaphrodites, n = 158; *aptf-1* hermaphrodites, n = 33; wild-type males, n = 120; *aptf-1* males, n = 35). Data was smoothed using a 10-minute moving average. Traces of wild-type hermaphrodites and males are the same as in Figure 1B.

(B) Duration of each developmentally timed sleep (DTS) episode for wild-type (orange) and *aptf-1(tm3287)* (light green) hermaphrodites. Statistical significance was evaluated using Mann-Whitney U tests with FDR correction. (L1 wild-type hermaphrodites, n = 152; L1–L3 *aptf-1* hermaphrodites, n = 33; L2 wild-type hermaphrodites, n = 155; L3 wild-type hermaphrodites, n = 157; L4 wild-type hermaphrodites, n = 156; L4 *aptf-1* hermaphrodites, n = 23).

(C) Fraction of time spent in quiescence during each DTS episode of wild-type (n = 147) and *aptf-1* (n = 23) hermaphrodites. Statistical significance was evaluated using Mann-Whitney U tests with FDR correction.

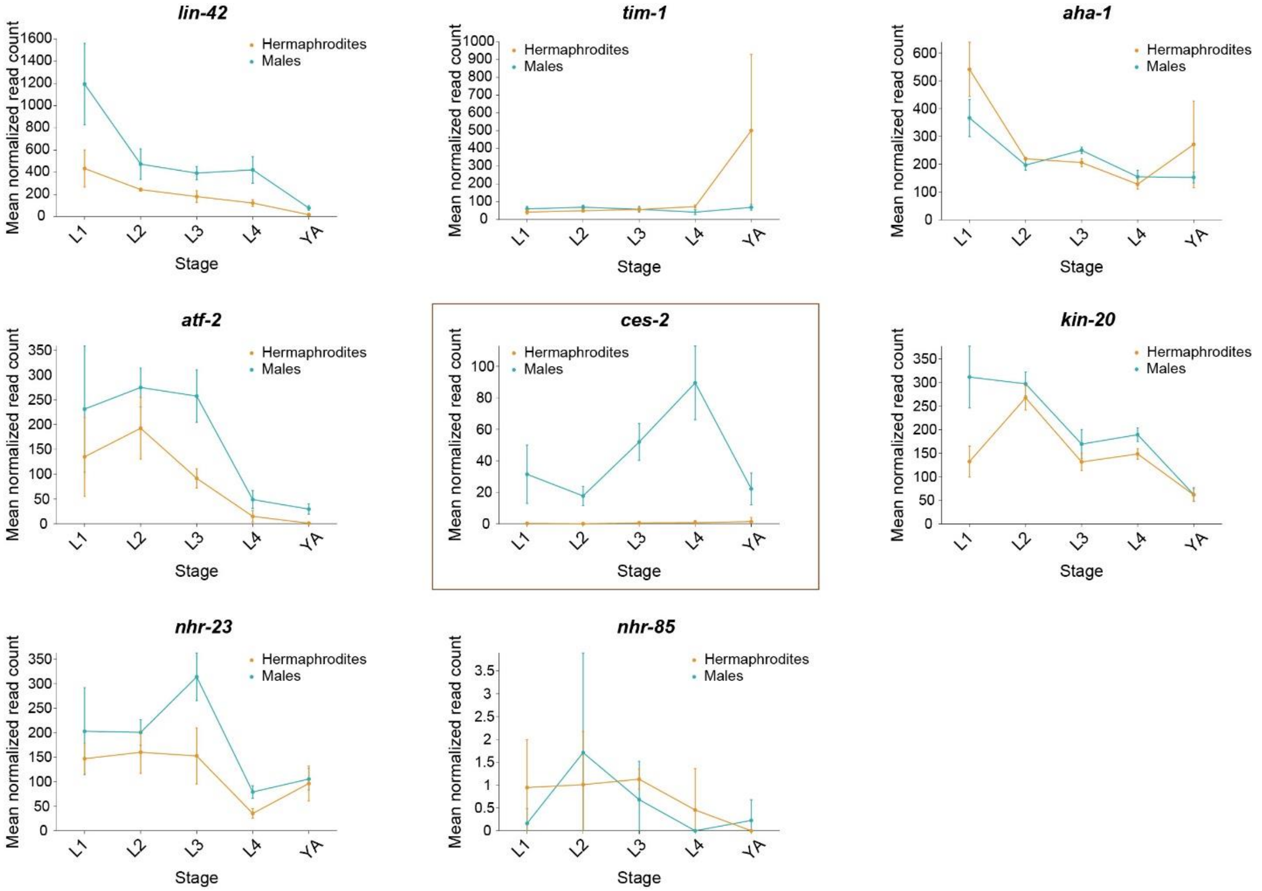

Figure S3. Gene expression profiles of clock gene homologs across development, related to Figure 3

Normalized RNA-seq expression values of clock gene homologs for males and hermaphrodites across all developmental stages, $n = 4$ biological repeats per sample. Expression data is taken from Dimorgena [S1].

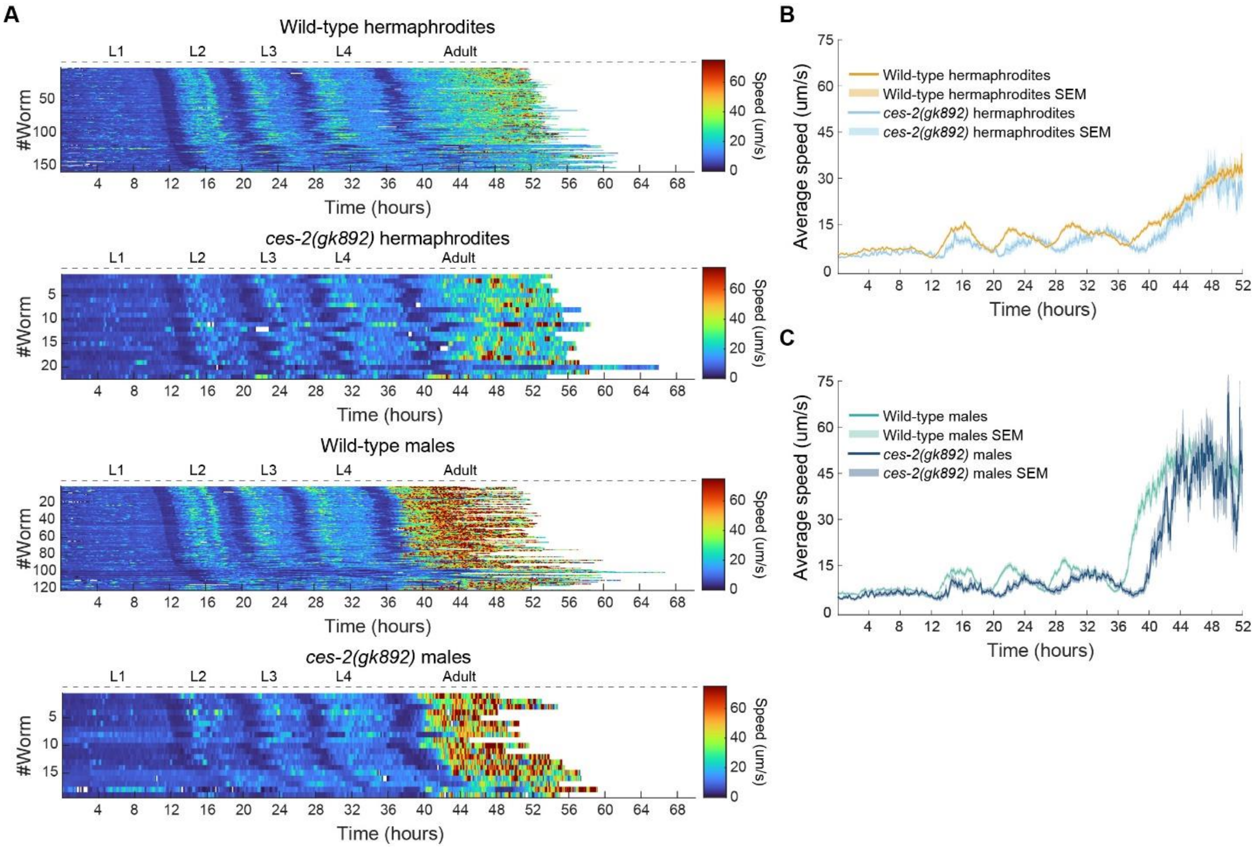

Figure S4. Elongated development of *ces-2(gk892)*, related to Figure 3

(A) Locomotion speed of wild-type and *ces-2(gk892)* hermaphrodites (top two panels) and wild-type and *ces-2(gk892)* males (bottom panels) from hatching until ~16 hours of adulthood. Traces are sorted by entry into the first molt. (wild-type hermaphrodites, n = 158; *ces-2(gk892)* hermaphrodites, n = 22; wild-type males, n = 121; *ces-2(gk892)* males, n = 19).

(B) and (C) Average speed across development of wild-type (orange) and *ces-2(gk892)* (light blue) hermaphrodites (B) and wild-type (turquoise) and *ces-2(gk892)* (dark blue) males (C). Shaded areas indicate ± SEM.

(A-C) Data was smoothed using a 10-minute moving average. Traces of wild-type hermaphrodites and males are the same as in Figure 1B.

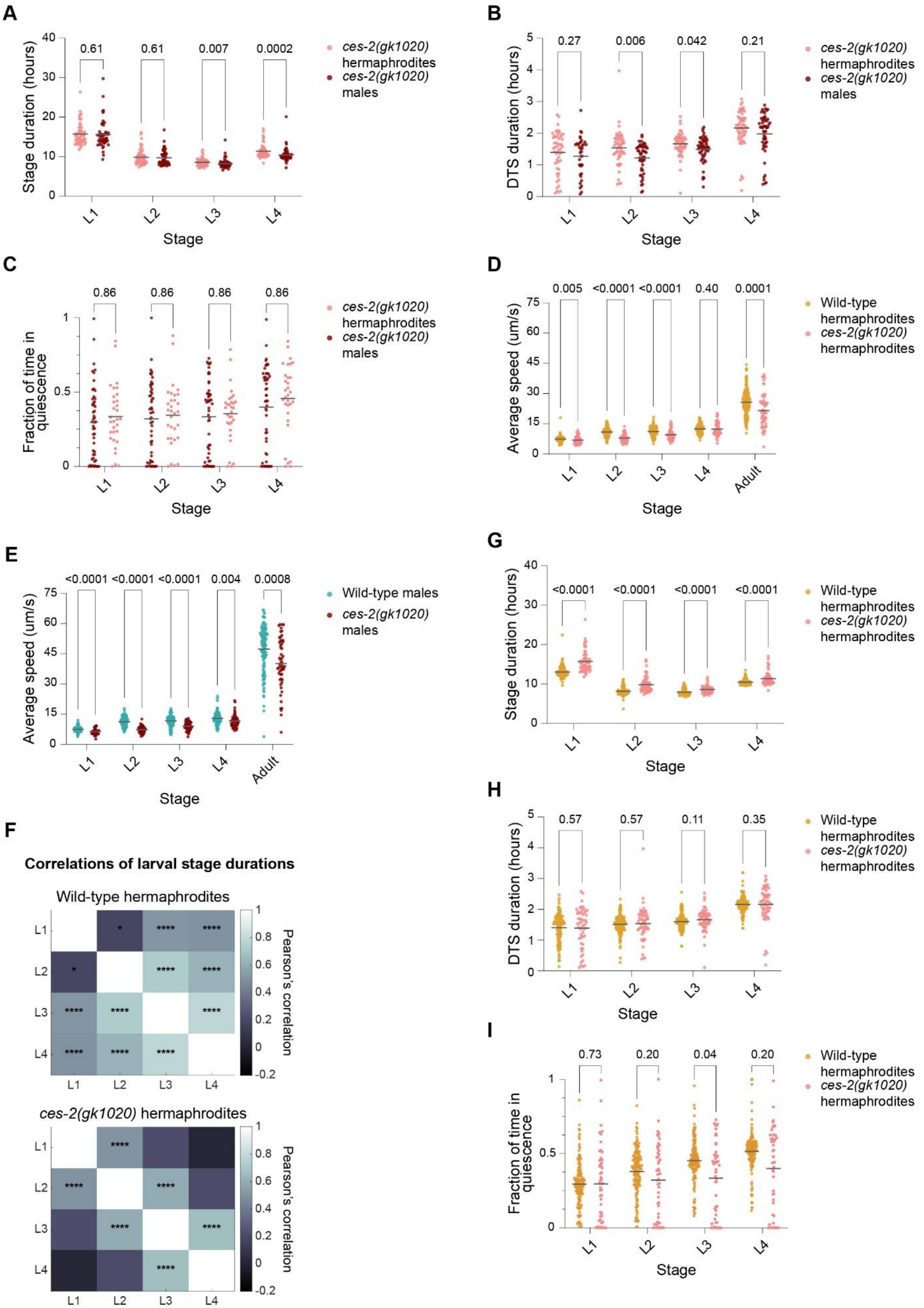
A
Stage duration (hours)
0.61
0.61
0.007
0.0002
L1
L2
L3
L4
Stage
ces-2(gk1020) hermaphrodites
ces-2(gk1020) males
B
DTS duration (hours)
0.27
0.006
0.042
0.21
L1
L2
L3
L4
Stage
ces-2(gk1020) hermaphrodites
ces-2(gk1020) males
C
Fraction of time in quiescence
0.86
0.86
0.86
0.86
L1
L2
L3
L4
Stage
ces-2(gk1020) hermaphrodites
ces-2(gk1020) males
D
Average speed (um/s)
0.005
<0.0001
<0.0001
0.40
0.0001
L1
L2
L3
L4
Adult
Stage
Wild-type hermaphrodites
ces-2(gk1020) hermaphrodites
E
Average speed (um/s)
<0.0001
<0.0001
<0.0001
0.004
0.0008
L1
L2
L3
L4
Adult
Stage
Wild-type males
ces-2(gk1020) males
F
Correlations of larval stage durations
Wild-type hermaphrodites
Pearson's correlation
L1
L2
L3
L4
ces-2(gk1020) hermaphrodites
Pearson's correlation
G
Stage duration (hours)
<0.0001
<0.0001
<0.0001
<0.0001
L1
L2
L3
L4
Stage
Wild-type hermaphrodites
ces-2(gk1020) hermaphrodites
H
DTS duration (hours)
0.57
0.57
0.11
0.35
L1
L2
L3
L4
Stage
Wild-type hermaphrodites
ces-2(gk1020) hermaphrodites
I
Fraction of time in quiescence
0.73
0.20
0.04
0.20
L1
L2
L3
L4
Stage
Wild-type hermaphrodites
ces-2(gk1020) hermaphrodites

Figure S5. Developmental timing, DTS, and locomotion in *ces-2(gk1020)* mutants, related to Figure 4

(A) Duration of each larval stage for *ces-2(gk1020)* hermaphrodites and males. Statistical significance was evaluated using Mann-Whitney U tests with FDR correction. (L1–L4 *ces-2* hermaphrodites, n = 57; L1 *ces-2* males, n = 50; L2–L4 *ces-2* males, n = 49).

(B) Duration of each DTS episode for *ces-2(gk1020)* hermaphrodites and males. Statistical significance was evaluated using Mann-Whitney U tests with FDR correction. (L1 *ces-2* hermaphrodites, n = 53; L1 *ces-2* males, n = 37; L2 *ces-2* hermaphrodites, n = 57; L2 *ces-2* males, n = 50; L3 *ces-2* hermaphrodites, n = 59; L3 *ces-2* males, n = 53; L4 *ces-2* hermaphrodites, n = 60; L4 *ces-2* males, n = 54).

(C) Fraction of time spent in quiescence during each DTS episode of *ces-2* hermaphrodites (n = 50) and males (n = 31). Statistical significance was evaluated using Mann-Whitney U tests with FDR correction.

(D) Average speed of wild-type and *ces-2(gk1020)* hermaphrodites in each developmental stage. Statistical significance was evaluated per developmental stage using Mann-Whitney U tests with FDR correction. (L1 wild-type hermaphrodites, n = 152; L1 *ces-2* hermaphrodites, n = 53; L2–L4 wild-type hermaphrodites, n = 153; L2–L4 *ces-2* hermaphrodites, n = 57; Adult wild-type hermaphrodites, n = 156; Adult *ces-2* hermaphrodites, n = 60).

(E) Average speed of wild-type and *ces-2(gk1020)* males in each developmental stage. Statistical significance was evaluated per developmental stage using Mann-Whitney U tests with FDR correction. (L1 wild-type males, n = 114; L1 *ces-2* males, n = 37; L2–L4 wild-type males, n = 108; L2–L4 *ces-2* males, n = 49; Adult wild-type males, n = 119; Adult *ces-2* males, n = 54).

(F) Correlation matrices of wild-type (top panel) and *ces-2(gk1020)* (bottom panel) hermaphrodites. Color indicates Pearson's correlations between every pair of larval stages. Asterisks represent the significance of each Pearson's correlation, based on two-tailed tests for nonzero correlation. Benjamini-Hochberg false discovery rate (FDR) correction was applied to control for multiple comparisons (see *Methods*). * $p < 0.05$; *** $p < 0.001$; **** $p < 0.0001$.

(G) Duration of each larval stage for wild-type and *ces-2(gk1020)* hermaphrodites. Statistical significance was evaluated using Mann-Whitney U tests with FDR correction. (L1 wild-type hermaphrodites, n = 121; L1–L4 *ces-2* hermaphrodites, n = 57; L2–L4 wild-type hermaphrodites, n = 119).

(H) Duration of each DTS episode for wild-type and *ces-2(gk1020)* hermaphrodites. Statistical significance was evaluated using Mann-Whitney U tests with FDR correction. (L1–L2 wild-type hermaphrodites, n = 121; L1 *ces-2* hermaphrodites, n = 53; L2 *ces-2* hermaphrodites, n = 57; L3 wild-type hermaphrodites, n = 123; L3 *ces-2* hermaphrodites, n = 59; L4 wild-type hermaphrodites, n = 121; L4 *ces-2* hermaphrodites, n = 60).

(I) Fraction of time spent in quiescence during each DTS episode of wild-type (n = 147) and *ces-2* (n = 50) hermaphrodites. Statistical significance was evaluated using Mann-Whitney U tests with FDR correction.

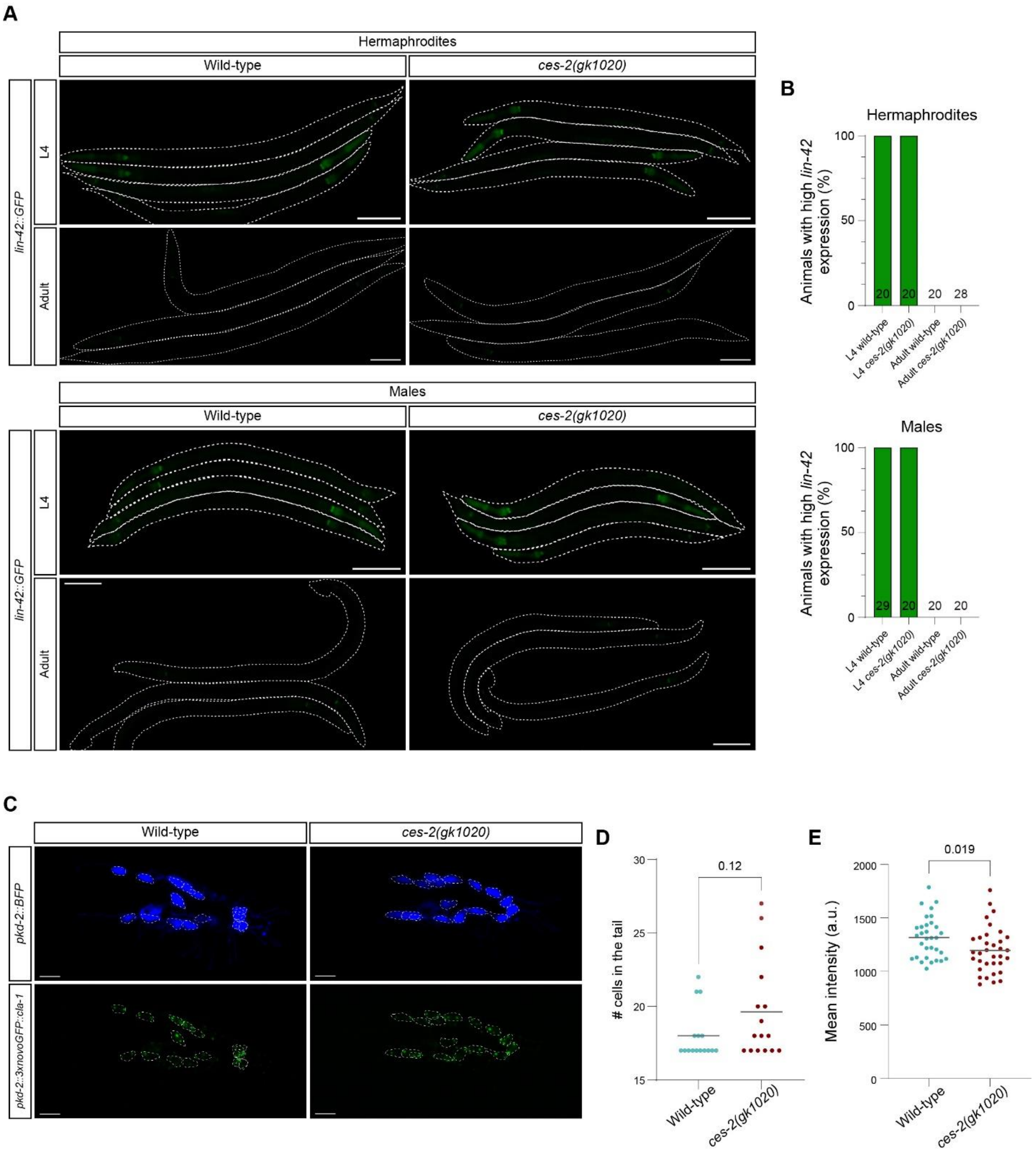

Figure S6. *lin-42* and *cla-1* expression in wild-type and *ces-2(gk1020)* animals, related to Figures 4 and 5

(A) Representative confocal micrographs showing *lin-42::GFP* expression in L4 and 1-day adult wild-type and *ces-2(gk1020)* hermaphrodites (top) and males (bottom). Scale bar, 100 µm. Worms are outlined with white dashed lines.

(B) Percentage of animals with high *lin-42::GFP* expression in the pharyngeal region. Numbers indicate sample size (n).

(C) Representative confocal micrographs showing *pkd-2::BFP* expression (top) and *pkd-2::3xnovoGFP::cla-1* expression (bottom) in the tail of 1-day adult wild-type and *ces-2(gk1020)* males. Scale bar, 10 µm. Cell bodies are outlined with white dashed lines.

(D) Quantification of the number of cells expressing BFP under the *pkd-2* promoter in the tail of wild-type (n = 16) and *ces-2(gk1020)* (n = 16) males. Statistical significance was evaluated using Mann-Whitney U test.
(E) Quantification of *pkd-2::3xnovoGFP::cla-1* fluorescence intensity for wild-type (n = 32) and *ces-2(gk1020)* (n = 34) males. a.u., arbitrary units. Statistical significance was evaluated using unpaired t-test.

Supplemental references

[S1] Haque, R., Kurien, S.P., Setty, H., Salzberg, Y., Stelzer, G., Litvak, E., Gingold, H., Rechavi, O., and Oren-Suissa, M. (2024). Sex-specific developmental gene expression atlas unveils dimorphic gene networks in C. elegans. Nat. Commun. *15*, 4273. https://doi.org/10.1038/s41467-024-48369-z.
